# Medicine

**Published:** 1900-06

**Authors:** R. Shingleton Smith


### Progress of the Medical Sciences.

### MEDICINE.

**Pneumonia** is the subject of numerous papers in the *Practitioner* (February, March, 1900), whose editor still adopts the praiseworthy practise of devoting the greater part of his monthly number to one topic. Unfortunately he has no new discovery to announce, and his efforts are not quite worthy of being spoken of as a campaign against the enemy; nevertheless the subject of pneumonia is one of perennial interest, and in which the possibilities of the future are as great as the needs of the present.

Dr. Julius Dreschfeld[2] sums up the details of prophylactic, specific, and symptomatic treatment, and advises that "as the micro-organism of pneumonia is contained in the sputum, this should be disinfected either by a 5 per cent. solution of carbolic acid or a solution of 1 in 1000 of corrosive sublimate." He further advises "that weak and elderly people suffering from bronchial catarrh should avoid the sick-room of patients suffering from pneumonia." Dr. R. W. Philip prefers the name

"pneumonic fever" to the more commonly accepted term "pneumonia," and remarks that "the physician is wisely guided who thinks of it less as a lung disease than as an acute continued fever of comparatively short duration with local manifestation, especially in the lungs. The local (pulmonary) lesion may be considered analogous to the intestinal lesion in typhoid, or the throat lesion in diphtheria or scarlatina. The local lesion is accompanied by systemic infection. The specific organism (the diplococcus pneumoniæ of Fränkel) has been discovered not only in the lungs but in the pleura, pericardium, endocardium, meninges, kidneys, spleen, and blood. There is abundant reason for the statement that the constitutional symptoms of pneumonia result from the formation by the diplococcus of a toxin—the so-called pneumotoxin. Such a toxin has been separated from cultures of the organism. If animals be inoculated with the toxin grave constitutional symptoms ensue, including pyrexia and circulatory disturbances. Thereafter, there would seem to be gradually developed an antagonising principle or anti-pneumotoxin, which may reasonably be expected in the near future to play an important part in the prevention and treatment of pneumonic fever."[1]

The difficulties in accepting the germ theory of the etiology of pneumonia are so great that much evidence is needful, and the arguments in favour of regarding the pneumococcus as the sole efficient cause of lobar pneumonia have been collated from French authors,[2] and "the constant presence of the pneumococcus of Talamon-Fränkel in lobar pneumonia, and the plurality of microbes causing broncho-pneumonia, are the distinctive elements which bacteriology permits us to recognise in the history of pulmonary inflammations (Netter)." This point has been further commented upon by Andrew H. Smith,[3] who remarks that "speaking generally, bronchopneumonia is shown to be associated with several varieties of pathogenic organisms; for example, the streptococcus, staphylococcus, Friedländer's bacillus, the tubercle bacillus, and others; but chief among them all is the pneumococcus, which, taking all cases together, is present either alone or in association with some of the others named, in at least fifty per cent. . . . The conclusions to which this evidence points are obviously these:—1. That the primary and secondary bronchopneumonias have a different bacteriological origin. 2. That secondary bronchopneumonia is for the most part due to streptococcus infection, derived from some source in connection with the air tubes, throat, or mouth. 3. That primary bronchopneumonia is of pneumococcal origin. 4. That pneumococcus inflammation occurs with almost equal frequency in the child and in the adult. 5. That pneumococcal inflammation takes a different form in each, in the adult producing massive consolidation and in the child disseminated

[1] *Practitioner*, 1900, lxiv., 265. [2] *Ibid.*, 164.
[3] *Twentieth Century Practice*, vol. xvi., 1899, p. 135.

patches of consolidation; in other words, that there are no real pathogenic distinctions between lobar pneumonia of the adult and primary lobular pneumonia of the child."

Dr. J. W. Washbourn[1] goes further into the question of pneumococcal infections, and finds that lobular pneumonia may be caused by several kinds of micro-organisms: the most frequent are the pneumococcus, the streptococcus pyogenes, and the influenza bacillus, and that we must "look upon pneumonia as a septicæmia, the inflammation of the lungs being simply a local attempt to shut in the cocci and prevent them from entering the circulation." The characteristics of influenzal pneumonia have recently been described by Dr. Whitla.[2] In his opinion the most remarkable and important characteristic of this form of pneumonia is its contagiousness, and this independently of influenza itself. It attacks equally those who have already passed through influenza, those suffering from it, and those who appear to have escaped.[3]

The paramount ascendancy of the germ is shown by the fact that lobar pneumonia is defined by Andrew H. Smith[4] as follows:—"An acute disease in which a specific parasite invades the air cells of one or more pulmonary lobes, where it grows in a fibrinous medium exuded from the functional capillaries, and generates a toxin that infects the system at large." He regards the disease, not as an inflammation, but as a germ culture, the medium for which is supplied from the functional vessels. "This structural immunity in an organ which is the seat of the most virulent disease is characteristic of pneumonia, and it is made obvious to us in the surprising rapidity of the process of recovery when the latter has once set in. For the lung to return to the normal state all that is wanted is a removal of the accumulated *materies morbi*."[5]

Dr. Ewart remarks[6] that "we have been accustomed to regard a short attack of pneumonia and a rapid clearing of the lung as much to be desired, and it will be difficult to convince us that we have erred in holding that belief. At any rate, we are indebted to Raven[7] for the novelty of his views on pneumonia and of the special construction which he places upon the clinical history of a case of pneumonia in a boy, aged twelve years, in which consolidation was rather delayed, but very rapidly disappeared—too rapidly, according to Raven—leaving in its train a long series of glandular and mediastinal suppurations. Raven regards consolidation as a conservative process. . . . He, therefore, deprecates the use of the ice-pack."

As regards the specific treatment, Dreschfeld points out that, although experiments in animals have shown that they can be rendered immune to inoculations with the pneumococcus, and

[1] *Practitioner*, 1900, lxiv. 272. [2] *Dublin J. M. Sc.*, 1899, cvii. 241.
[3] Quoted in *Practitioner*, 1900, lxiv. 325.
[4] *Op. cit.*, p. 3. [5] Quoted in *Progressive Medicine*, 1899, iii. 45.
[6] *Ibid.* [7] *Practitioner*, 1898, lxi. 269.

the blood serum of such animals has also been found to possess protective properties, and these facts have been made use of for the treatment of pneumonia in man, yet, "with the data at present available, it is difficult to arrive at a definite conclusion as to the value of the treatment."[1] Protective serum is now supplied by Dr. Pane, of Naples; it is administered by subcutaneous injection, in the same manner as the diphtheria antitoxin. The dose is from ten to twenty cubic centimetres, which may be given two or three times a day during the attack, until the constitutional symptoms have commenced to subside.

With reference to the method which has been described as jugulation by means of large doses of digitalis, it is singular to note how few physicians have had the courage to carry out Petrescu's method to the full; nevertheless "digitalis in recent years has held a prominent place in the treatment of pneumonia. With the recognition that the chief danger is from heart failure, it is natural to expect benefit from the use of a drug that is of such great service in cardiac diseases in restoring the rhythm of the contractions, reducing their frequency, and giving them greater force and efficiency."[2]

In spite of these considerations, the author of this quotation adds: "With my present views I consider the use of the drug in pneumonia without the simultaneous employment of an arterial dilator a highly dangerous and indefensible measure, and one that has contributed in no slight degree to the fatality of this disease."

Dreschfeld has used a fresh infusion of digitalis (20 grains to 6 ounces), and has given half an ounce every two hours. The writer has frequently given half-ounce doses of the ordinary pharmacopœial infusion every hour, and has not observed any unfavourable result, although he has not obtained effects worthy of being spoken of as jugulation. The routine treatment with tincture of digitalis and chloral, so highly recommended by Balfour, was found by Dreschfeld to have a good effect in overcoming the insomnia.

Dr. W. H. Thomson, commenting on the therapeutics of heart diseases,[3] remarks on digitalis: "The general effect of its action, however, is to diminish the size of the heart's cavities. The drug, therefore, is of the greatest service when the heart walls are over-dilated and too much residual blood remains after each systole from inability of the dilated cavities to contract strongly enough to expel their contents. . . . But here comes the important fact about digitalis, that in order to produce its contractile effect it must have a more or less normal muscle fibre to work upon. It is utterly powerless with degenerated heart muscle." Hence the drug would appear to be just the thing needed in pneumonia, where the heart-muscle is not commonly degenerated, but the heart-walls are commonly dilated.

[1] Dr. Washbourn, *Practitioner*, 1900, lxiv. 252.

[2] Andrew H. Smith, *op. cit.*, p. 115. [3] *Med. Rec.*, 1900, lvii. 442.

It is of interest to observe that Maragliano attributes to digitalis a specific action on the pneumococci, which are killed by a very small quantity of it, while the toxin of pneumonia when injected is also found to be partly neutralised by digitalis. He thinks that this peculiarity is an explanation of the fact that pneumonic patients will stand larger doses of digitalis than healthy persons.[1] Eustace also holds that digitalis is antagonistic to pneumonia, and that sufficient should be given to slow the pulse. Alcoholic stimulants tend to inhibit this specific action, and he feels sure that he gets better results by combining ammonia with digitalis.[2] The writer finds it is difficult to produce digitalis symptoms in patients with pneumonia. It seems to be almost impossible to give too much so as to destroy both the cocci and the patient.

The frequent occurrence and fatality of pneumonia amongst our troops in South Africa at the present time gives an especial interest to the views of Dr. Alfred Hillier on Dust Pneumonia. The occurrence of chronic interstitial pneumonia, and of the varieties of "pneumokoniosis" described by Arlidge,[3] have long been recognised, and have been looked upon as often the forerunners of phthisis; but it is now suggested that dust may be capable, under certain conditions, of exciting an acute inflammatory condition of the lung—the presence of pneumococci assisting the mechanical irritation of dust particles in bringing about this result. The statistics quoted by Dr. Newsholme[4] seem to support this view. The cloud of dust raised by the automotor car, and the necessary inhalation of much dust in every day cycling, should give us increasing evidence in this direction.

Some papers on the bacteriology of Epidemic Pneumonia in South Africa are quoted in *Progressive Medicine*.[5] An epidemic, with a mortality of 60 to 70 per cent., had raised the suspicion that the South African pneumonia was of a different type from that of the European disease. Kolle,[6] however, has demonstrated their identity. In 15 necropsies the diplococcus of Fränkel was found in 11, and the influenza bacillus in 4, and an examination of the sputum gave analogous results. Similar conclusions are reported to us from the Transvaal. The authors conclude that: (1) The diplococcus described is identical with the pneumococcus of Fränkel, but possesses greater virulence; (2) The pneumococcus is the causal factor of cerebro-spinal meningitis; (3) The pneumococcus affects the nasal mucous membrane first with rhinitis, leading to subsequent infection of the frontal and nasal sinuses, of the middle ear, of the parotid, of the lung, and of the meninges; while a pneumococcic septicæmia, fatal in itself apart from pneumonia or meningitis, may develop as a result of the pneumococcic rhinitis.

[1] *J. Am. M. Ass.*, 1898, xxxi. 472. [2] *Brit. M. J.*, 1898, i. 1656.
[3] *The Hygiene, Diseases and Mortality of Occupations*, 1892.
[4] *Practitioner*, 1900, lxiv. 46.
[5] 1899, iii. 47. [6] *Deutsche med. Wchnschr.*, 1898, xxiv. 425.

Another variety—the so-called Ether Pneumonia—is described by Dr. J. F. W. Silk.[1]

The occurrence of leucocytosis in pneumonia has received much attention of late, and Dr. Alfred Stengel[2] has recently reviewed and enlarged our knowledge thereon. Dr. Hector Mackenzie[3] remarks that "at present it cannot be said that an examination of the blood and a count of leucocytes in a case of pneumonia are of great practical importance. We usually possess, independently of such an examination, sufficient indications of the severity of a case of pneumonia." The method of treatment founded on the encouragement of leucocytosis, *i.e.*, the promotion of local suppuration by the injection of turpentine subcutaneously, does not appear to have met with much favour. Dr. Stengel, who tried it in one case, "thinks that the good result which followed may have been due to the local relief afforded by the abstraction of large numbers of leucocytes from the lungs rather than to phagocytic influence."

The following is a *résumé* given by Dr. Andrew H. Smith:[4] "In the view of the writer, the treatment of pneumonia should embrace the following points:

"An attack upon the pneumococcus through the medium of the blood, the object being that the exudate when it escapes into the air cell shall be impregnated with a substance that will unfit it to serve as a culture medium.

"Stimulation of the emunctories to throw off the poison as it forms.

"Sustaining the vital powers and particularly the heart—cardiac stimulants.

"Relieving the pulmonary circulation—vasodilators, venesection.

"Compensation for loss of respiratory surface—inhalations of oxygen.

"Reduction of excessive temperature—cold to surface, antipyretics (?).

"Relief of incidental symptoms."

R. SHINGLETON SMITH.

[1] *Practitioner*, 1900, lxiv. 307. [2] *J. Am. M. Ass.*, 1899, xxxiii. 438.
[3] *Practitioner*, 1900, lxiv. 323. [4] *Op. cit.*, p. 122.

